# The Royal Chest Hospital Scandal

**Published:** 1889-10-26

**Authors:** 


### THE ROYAL CHEST HOSPITAL SCANDAL.

### A STATEMENT OF THE FACTS.

The following is the statement of the matters in dispute, as read by order of the council, to the special meeting of governors, held on October 7th, 1889. So much interest has been aroused and the issues at stake are so momentous to the interests of our voluntary hospital system that it has become advisable to print the facts so far as they are at present available:

### The First Charges against the Secretary.

During the early part of last winter it came to the knowledge of the President and chairman of council that the action of the secretary to the council had, in certain particulars, been such as seriously to affect the interests of the hospital. Fully realising the gravity of the statements they had heard in respect of an officer holding so important a position at the hospital, they were anxious to show Mr. Austin every consideration, being mindful of the services he had rendered to the institution. Accordingly, in the first instance, they took upon themselves to enquire as fully as possible into such statements, and they gave the secretary every opportunity of replying to the same. As the result of their investigation, they came to the conclusion that they had no other alternative but to report to the council the course they had taken. This they accordingly did at the meeting of the council of February 25, and a committee was appointed to consider certain charges brought against the secretary, consisting of the following members: The treasurer, the chairman and vice-chairman of council, the chairman and the treasurer of the building committee, the chairman of the house and finance committees, the chairman of the medical council. At the first meeting, March 7th, the treasurer was elected chairman, and it was decided that Mr. Austin should be present at the meetings in order that he might hear the allegations brought against him, and that every facility might be granted him for replying to the same. Before proceeding to consider their report, the committee afforded Mr. Austin the opportunity of calling any witnesses he might name, and also gave him ample time to prepare a written reply to the allegations brought against him. He accordingly called several witnesses, who appeared before the committee on his behalf.

### The Secretary's Charges against the Medical Staff.

Having read that part of his reply which related to the allegations which had formed the subject of the committee's enquiry, Mr. Austin at once proceeded, in such written reply, to make certain statements reflecting upon the conduct of the medical staff. In expressing his belief as to the causes of the continual opposition and lack of co-operation which he had received from some of the medical staff, he said: "Added to this, has been the knowledge that I have, from time to time, thought it my duty to watch carefully the increasing tendency to make the hospital less a hospital for consumption for the treatment of poor patients recommended by subscribers, than a school for the education of the doctors. Many persons recommended by subscribers are refused admission as in-patients, while the number of persons admitted as 'emergency cases' has been very large, nearly sixty such cases having been brought here during the present year. Many of these are, I believe, brought without any hope that they will live to leave the hospital. Sometimes they are here for months without anything being contributed directly or indirectly towards their support. One of these cases I submitted to the council some time ago. It was that of a poor child, who was placed in the Helena Ward by herself. Special nurses were engaged for her, and then the house physician had the opportunity of performing his first tracheotomy operation. The poor child died soon after." The minute-book gives no record of this case having been submitted to the council. It was, however, reported to them by the secretary, Nov. 12, 1886, merely as a case which required the engagement of special nurses, for which sanction was given. Mr. Austin went on to say: "Another of these emergency cases was that of a strong man of 48, afflicted with aneurism, but who on his admission said he felt quite well. After being in the hospital for some weeks, the experiment of introducing 32 feet of steel wire into him was tried, with the result that he died two or three days after. I mention these instances, not as in any way pretending to offer an opinion as to the medical treatment of the patients in the hospital, but as being anxious to maintain its reputation as an institution founded for the benefit of the poor of the neighbourhood, and supported mainly by local subscribers, employers, and working-men." Mr. Austin ended thus: "In what I have said above, I have purposely abstained, as much as possible, from personal allusions, my wish being that all the officers, whether honorary or paid, should work together in subordination to the council for the good of the hospital."

Decision of Special Committee and the Council.

This concluded the secretary's reply, and at their sixth sitting, on May 13, the committee drew up their report, which simply recorded their decisions on the subjects for investigation which had been submitted to them without any recommendation to the council as to the action which should be taken thereon. The report was presented to the council at their next meeting on May 29, at the conclusion of the ordinary business on the agenda. After a general statement of the proceedings of the committee and the reading *in extenso* of the latter part of Mr. Austin's reply, a resolution was proposed and seconded to the effect that the secretary should be called upon to resign. To this the following amendment was moved, seconded, and carried: "That this council is of opinion that in several matters reported to it by the committee the secretary has acted in a manner not calculated to ensure the best interests of the hospital, and is hereby severely censured for such conduct." This resolution was at once communicated to Mr. Austin.

Action taken by Medical Staff.

On June 3rd the following letter was addressed by the honorary medical staff to the chairman of the council, who, in accordance with law xi., 2, summoned a special meeting of the council on June 27th to consider the same.

"We have received from the president of the hospital a copy of the reply made by the secretary to the council to certain charges against him which have been recently investigated by a sub-committee of council appointed for that purpose, which reply was laid upon the table at the meeting of council held on the 29th of May, 1889. Our attention has been specially called to the latter part of section v., [printed above], which was read in full at the above meeting, and which raises matter entirely foreign to the enquiry before the sub-committee. We can readily understand that the full import of these paragraphs may not have been duly appreciated by the members of the council present, especially as they were read towards the close of a prolonged sitting. But we would point out that they contain certain distinct statements and innuendoes reflecting in the gravest manner upon the professional conduct of your medical staff. These charges, however, do not, in our opinion, merely affect the medical staff, but seriously compromise the character of the hospital as a charitable institution, appealing constantly to the public for support. We have, therefore, to request that your council will immediately investigate the statements of the secretary to the council, above quoted. Our sense of their gravity compels us at the same time to tender to you, unreservedly, our resignation of the offices we hold in your hospital. We, further, as members of the medical council, request that, in accordance with law xi., section 2, you will summon a special meeting of the council at the earliest date to consider this letter and to investigate these charges. We have the honour to remain, etc., etc."

(Signed) Philip J. Hensley, M.D., F.R.C.P., Senior Physician to the Hospital; T. Gilbart Smith, M.D., F.R.C.P., Physician to the Hospital; David W. Finlay, M.D., F.R.C.P., Physician to the Hospital; Wm. Henry White, M.A., M.D., M.R.C.P., Physician to the Hospital; Oswald A. Browne, M.A., M.B., M.R.C.P., Physician to the Hospital; Arthur T. Davies, B.A., M.B., M.R.C.P., Physician to the Hospital; S. Herbert Habershon, M.A., M.D., M.R.C.P., Assistant Physician to the Hospital; Edward Stewart, M.D., M.R.C.P., Assistant Physician to the Hospital; James Calvert, M.D., M.R.C.P., Assistant Physician to the Hospital; A. Pearce Gould, M.S., F.R.C.S., Surgeon to the Hospital.

Proceedings of the Council.

The letter from the medical staff having been read, it was decided that Dr. Hensley and Dr. Finlay should be present. The secretary stated that he considered his reply read before the committee was a privileged communication, and that, as it had been dealt with by the council, it was at an end. He denied that he had made any charges against the medical staff, having stated in his reply that he did not offer an opinion as to the medical treatment of the patients. His statement related mainly to the financial position of the hospital. Dr. Hensley, as representing the honorary medical staff of the hospital, then read a general reply to the allegations brought against his colleagues and himself by Mr. Austin in his letter of May 13, of which the following is the substance:

Medical Staff's Reply.

Dr. Hensley began by reading a letter which he had written to Lord Charles Bruce, in the course of which he explained the feelings which had prompted him and his colleagues to take the course of action they had done. It stated:

"The paragraphs quoted in our letter to Mr. Morley appear to be intended to mean this, if anything at all: That I have abused my position at the hospital by causing to be excluded from its benefits fit applicants, and by causing to be admitted these patients, not with the desire and hope of doing all that lay in my power, but for the purpose of recklessly making experiments upon them, and that the deaths of such patients were due to my recklessness. Now were there any truth in this I should be unfit to hold the office I do, and it would be the bounden duty of the hospital authorities to take immediate steps to oust me. I do not, however, admit that I am in the smallest degree guilty of what is thus imputed to me by an official of the hospital, and yet I should be placed in the position, if I remained in office as physician, of submitting in silence to have such things said of me. I do not believe that the council, either collectively or individually, can really think that there is any foundation for the charges insinuated by the secretary; but I would point out that such charges obtain a degree of countenance from the fact that they have been brought under its notice without eliciting any special remark." Dr. Hensley then proceeded to take point by point what was asserted or implied in Mr. Austin's letter, and to supply information as to the truth on all matters erroneously exhibited therein.

The True Aspect of Matters.

The substance of what he said may be put under six heads, as follows:

*(1) Stated or implied by the language of Mr. Austin: That the number of patients suffering from consumption admitted to the hospital (which he calls a "hospital for consumption") has been unduly restricted.*

(1) A. The hospital is not one exclusively or mainly for the treatment of consumption. It is known as the Royal Hospital for Diseases of the Chest, and thus professes to treat all forms of chest disease, and makes no promise to admit one class of such diseases in numbers out of their due proportion. B. As a matter of fact, the number of cases of consumption admitted has been 51 per cent. of the whole number admitted whereas, according to the Registrar-General's returns, the proportion cases of consumption to all chest disease is only 26 per cent. It thus appears that the number of cases of consumption admitted has been nearly double what might have been anticipated.

*(2) That the medical staff have proceeded as if the hospital existed rather for the doctors and their education than for the patients.*

(2) A. No medical man can properly perform his duties in a hospital without thereby constantly gaining experience and knowledge. So in a proper sense a hospital is and must be a "school for the doctors," and the better the work of a hospital is done—the more for the benefit of the patients—the better will it be as a school of experience for the doctors. B. The suggestion that in an improper way the hospital has been made a school for the doctors, which occurs in what is said about the two operations, will be noticed immediately.

Suitable Patients Not Refused Admission.

*(3) That many persons recommended by subscribers are refused admission as in-patients.*

(3) A. No one is thus refused unless found on examination by members of the medical staff to be an unsuitable case. B. It is part of the duty of the members of the medical staff to discriminate between the suitable and the unsuitable, and there are rules and notices relating to this point printed on the letters, which run thus: "In order to secure the greatest amount of good to the largest number of patients and to prevent the hospital being made an asylum, cases of consumption can be admitted only in the early and curable stages or when complicated with symptoms admitting of relief. No contagious or infectious disease, no surgical case, and no obviously hopeless case will be admitted." Again: "If a patient, applying at the hospital with a governor's letter for an in-patient, be found by the physician in attendance to be an unsuitable case for admission to the wards, or if from any other cause it cannot be admitted, the patient will be given an out-patient's letter, and the in-patient's letter will be forwarded to the recommending governor."

Emergency Cases Not Admitted in Excess.

*(4). That emergency cases (described as persons brought to the hospital for some of whom nothing is directly or indirectly contributed) have been admitted in excess.*

(4) A. All these cases come to the hospital recommended by subscribers, and therefore it cannot be said that nothing is contributed for them. B. They cannot be said to be brought to the hospital in any other way than are those who come to the hospital with in-patient's letters, and (as an examination of the addresses show) they come in somewhat larger proportion from the parishes immediately surrounding the hospital. C. They come with out-patient letters (thus recommended by subscribers, as are those who come with in-patient letters), and it is part of the duty of the physicians to recognise when the condition of their patients is such that admission in this way is desirable for them. There is a notice in reference to this matter printed on the letters, which runs thus: "Acute cases are admitted at once upon the advice of a member of the medical staff." D. For many of those thus admitted, in-patient letters might readily be obtained after admission: this is a matter which does not fall within the province of members of the medical staff, but of the secretary who, although his attention was specially called to it by the resident medical officer, appears to have taken no steps thereon.

No Hope of Recovery.

(5) *That of those admitted thus (as emergency cases), a large number are admitted without hope of their living to leave the hospital.*

(5) A. An examination of the numbers shows that: The mortality among the cases admitted as emergencies is less than among those admitted with in-patient letters. B. Though there may be little or no hope that a patient when admitted may "live to leave the hospital," it may yet be the very best thing that such a patient be admitted. It is scarcely less the function of a hospital to alleviate the suffering of the dying than to treat those who have a prospect of recovery.

### The Two Cases Mentioned.

(6) *Two cases are particularised and mentioned in a way to imply: A. That they were admitted rather for purposes of experiment by the doctors, than treatment for their benefit. B. That they were improperly made subjects of experiment while in the hospital. C. That their deaths were due to such treatment.*

### The Case of Tracheotomy.

(6) The first of these cases was a child named Edith Jacob, three years old, of 12, Hosier-lane, City, who was brought to the hospital with an out-patient's letter obtained from the Guildhall. After being seen once or twice as an out-patient by Dr. White, she was sent in as an emergency case, being then getting into a dangerous condition, though it could not be said with any truth that she was admitted without any hope that she would live to leave the hospital. On the contrary, there was a very reasonable hope; whereas it might be said that there was very little chance that she would live long with only such treatment as was possible for her outside a hospital. When admitted the child became my patient. In a sense you may say that this case was "submitted to the council." But in what sense? Solely as I understood at the time as to the expense involved by the extra nursing. It is now mentioned in a way to imply that improper treatment was then suggested, and that all this time I have been resting under this imputation. I will relate to you the history of the case while under my care: Immediately upon my seeing the child I said to the house physician (Mr. Simmons) that in all probability the operation of tracheotomy would shortly be necessary to give relief, and that after this the constant attention of a nurse would be for a time required. I enquired whether this could be managed, and he informed me that there would be no difficulty. I then gave him the following instructions: That if the difficulty of breathing became more urgent, and the necessity of the operation seemed to him to be becoming imminent, he was to send for me (if there were time) to finally decide, but that if in his judgment the urgency should be such as not to allow of time for this, then he was to perform the operation without delay. Such urgency did not occur, but on one of my visits I decided that it was best that the operation should be performed, and Dr. White, with whom I had a consultation, concurred in my opinion. Accordingly Mr. Simmons, under our direction and with our assistance, operated. Mr. Austin states that he then performed this operation for the first time. Whether this is so or not I do not know; what, however, I do know is this, that he did it very well. [This has since been proved not to be Mr. Simmons's first case of the kind.] I have seen this operation performed very many times by many different men, and I have seldom seen it better done. Bear in mind also that I was not in the least tied to allow Mr. Simmons to operate, but that if I had any reason to anticipate any special difficulty, or the slightest doubt of his competency (or even without either), I might have called in our surgeon, Mr. Gould. The operation was completely successful, and immediate relief was given to the breathing. After the operation she did well at first, and for a time we had fair hopes of her, but she was of a weakly constitution, and after a few days there was the addition of some bronchitis, under which she sank. An examination post-mortem showed that the only hopeful way of treating it for the obstruction which was found to exist was that which had actually been done. I believe that in every point what was done was done for the best, and in similar circumstances exactly the same would have to be done again.

### The Case of Aneurism.

The other patient mentioned is a man named John Raper, aged 48, a smith, of 38, Englefield-road, Hackney, who came to the hospital with an out-patient letter from Mr. S. Barnett, of 37, Alwyne-road, Canonbury. He was in a condition of extreme danger while he was going about, and Dr. White, whose patient he became, at once advised his admission. He had been a strong man, but to speak of a "strong man afflicted with aneurism," is much as if you were to say sound as a cracked bell. He may have said (as Mr. Austin states) that "he felt quite well." It is a remarkable fact that persons with large aneurisms very frequently do feel quite well—indeed, you might often say that barring the aneurism they are quite well. But in what sense can they say they feel quite well? In the same sense that the poor people of Johnstown, in Pennsylvania, said they felt quite safe just before they were overwhelmed by the bursting of the dam of the reservoir above their heads. The object of the operation (to put very shortly what was fully explained to the council) was to strengthen the wall of the aneurism (or saccular bulging of the large artery close to the heart), in the same way that the dam of the reservoir might have been strengthened by throwing in material to form a solid concretion on its inner side. And it is essential to the idea that there should be a considerable length of wire introduced, wire of a fineness such as that which is used for tying up flowers. The operation is spoken of as an experiment by Mr. Austin. To call baldly what was done an experiment, may tend to prejudice the minds of the hearers, but it might be quite fairly said that to a certain degree every operation or every dose of medicine administered is an experiment, and so would letting a thing alone be an experiment, and often an exceedingly dangerous one. Under what circumstances was the operation performed in this instance? The man had been kept quite quiet in the hospital for some five or six weeks, and under different treatment, but was getting worse, and it became evident that before long there must be a rupture, which would be followed by almost instant death. Dr. White then carefully explained to him the position in which he was: that if left alone he would be certain to die shortly from the sudden rupture, that the operation proposed involved in itself a certain amount of danger, that there was an uncertainty whether the desired effect would be produced, but that it did hold out a certain amount of hope of a favourable effect, and of a great prolongation of life. The patient elected to have it done, and accordingly it was done by Mr. Gould. Present: Drs. Hensley, Finlay, White, and Pringle. This case is reported in vol. lxx. of the Royal Medical and Chirurgical Society's *Transactions*, and the opinions of those competent to give them are there stated at length (p. 277, *et seq.*).

### Dr. Hensley Sums Up.

Dr. Hensley concluded with the following words: "What is the position of the medical staff of a hospital? There is a first necessity that we should endeavour to do our work well, conscientiously, honourably, to the best of our ability towards the good of the patients; that we should, in fact, deserve your confidence. But that is not enough unless we actually have your confidence. Nor is even this enough if it be not known that we have it, because almost as much harm would be done to the hospital if it were doubted whether we had your confidence as is if we were actually undeserving of it. And it may fairly be said that the expression of your confidence is wanting when it is possible for an official to make such statements as he has done without any special notice having been taken of it. Not to notice it, not to discountenance it in the most positive manner is at the very least to countenance it in some degree, and to place us in a position which is intolerable. It is with such feelings that we have taken the course we have. Therefore it is that we demand a definite expression of opinion from your council as to the truth or falsehood of the above charges, and this we have a right to expect, not only in our own interests and that of our profession, but in that of the patients, the governors, and the council itself."

### The Council's Decision.

Mr. Austin was heard in answer, and then, at the request of the chairman, Dr. Hensley, Dr. Finlay, and the secretary retired. The following resolution was then proposed, seconded, and passed by the council without a dissentient voice: "The council, having fully enquired into the very grave charges which have been brought by their secretary, Mr. Austin, against the medical staff of the hospital, are unanimously of opinion that such charges are utterly without foundation. They desire to record their sense of entire confidence in the members of their medical staff, fully assured that by their kindness and care, no less than by their skill, they have done their utmost to relieve the sufferings of the patients committed to their charge, and they deeply regret that allegations most seriously affecting their character and position should have been so recklessly made by an official holding so important a position at the hospital. The two cases specially referred to by the secretary are, to say the least, ill-judged. In the first he endeavours to bias laymen unfavourably with reference to the details of treatment in a case of extreme urgency, of which details he must necessarily be totally ignorant. In the second case, one of the most insidious ailments with which anyone can be afflicted, is made use of for the purpose of throwing doubt on the skill of those who anxiously hope and endeavour to alleviate the sufferings of their fellow creatures, be they rich or poor, and, further, in this particular case experience has warranted and justified the operation alluded to, if justification is necessary. The council consider the course of action taken by Mr. Austin perfectly unjustifiable, and that he has thereby gravely jeopardised the interests of the hospital. They, under these circumstances, can no longer retain his services as secretary to the hospital, and therefore call upon him to resign his office." The resolution was at once communicated to Dr. Hensley, Dr. Finlay, and Mr. Austin. The following resolution was also moved, and seconded, and carried without a dissentient voice "That in view of their possessing the full confidence of the council, the medical staff be requested to withdraw their resignation."

### The Secretary Resigns.

At the next meeting of the council, on July 8th, the secretary having retired, the minutes of the special meeting of the council of June 27th were read and confirmed, and the following letter from him, addressed to the chairman of the council, was read:

"In submission to the resolution passed at the special meeting of the council, held on 27th June last, I beg to tender to you my resignation of the office of secretary, which I have held since 13th November, 1884. But as I am advised that, under the general terms of my appointment, it should only be terminated at the same period of the year at which it was made, I submit that such resignation should not take place until the 12th of November next.

"While thus submitting myself to the decision of the council, I may I hope be allowed to protest that during the whole of the time that I have held the secretaryship I am conscious of having been actuated by no other motive than an earnest desire to uphold the authority of the council, as well as to promote in every way the highest interests of the charity.

"I must also beg to be allowed to repeat that, while offering no opinion as to the medical treatment of the patients, I am convinced that there is an increasing tendency to make the institution less a hospital for the treatment of poor patients recommended by subscribers than a school for the education of the doctors.

"1st July, 1889. (Signed) "John J. Austin."

The following resolution, duly moved and seconded, was then adopted:

"That Mr. Austin's resignation be accepted, and that his duties cease on October 1st, 1889, being three months from the date of his letter."

The Medical Staff Resume Office.

The following communication from the medical council was then read by Dr. Hensley: "At a meeting of the medical staff of the Royal Hospital for Diseases of the Chest, held at 10, Queen-street, on Friday, July 5th, 1889, the following resolution was proposed by Mr. Pearce Gould, seconded by Dr. White, and carried unanimously: 'The members of the medical council having received through the chairman a copy of the resolution carried by the council of the hospital at a special meeting held on 27th June, beg to return to the council their most cordial thanks for the assurance of confidence in them and appreciation of their services thus most gracefully and generously expressed to them. The difficulty of their position, which led to their placing their resignations in the hands of the chairman being now removed, they gladly withdraw their resignations, and desire to assure the council that no effort shall be wanting on their part which may tend to promote both the efficiency and economical working of the hospital, whether it be by means of their more usual medical duties, or by such consultation and co-operation with the council and other committees as may be most helpful to those objects. And it was resolved that the same should be entered on the minutes.'" Mr. Austin returned to the board-room and was informed of the action taken by the council during his absence, and the resolution which had been passed and the communication from the medical council were read to him. The council then adjourned.

Subsequent Proceedings.

On the 11th September, a meeting of the governors was held to authorise the sale of some of the hospital funded property to defray the cost of the new wing, etc. The secretary's friends attended in force, and although the resolution was declared to be carried, the president, the treasurer, and the chairman of council determined to take no action until they had afforded the governors a further opportunity of expressing their opinion. Accordingly a special meeting of the governors was held on October 7th, when the sale of £6,000 of funded property was sanctioned, and a resolution was also passed by 57 votes to 22 requesting the council to reconsider the resignation of the secretary, although he persisted in reasserting his views as expressed above. (*Vide* The Hospital for October 12th, pp. 31 and 32.) The council held a meeting on the same evening, and resolved that no grounds existed for altering their decision respecting the dismissal of the secretary. Thereupon, the secretary's friends assembled at the Spencer-place Chapel, Goswell-road, on the 11th October, "censured the council and pledged themselves to further action," and further "resolved that a requisition be signed by twenty governors calling upon the council to immediately call a meeting to take such steps as shall ensure the peaceable working of the hospital in the future." The chairman of this meeting, Mr. George Berry, is reported to have then said "that their position was that the confidence of the governors was broken in the council, and they were therefore justified in calling the meeting and censuring the council." Since then the chairman of the council, Mr. S. Hope Morley, with reference to these resolutions and statements, has written to ask all impartial persons "to withhold their final judgment upon the merits of the dispute," because he "has the strongest confidence that when the whole of the circumstances are made known, as in due course they will be, the public will not long hesitate to say upon whose shoulders must rest the responsibility for a course which is doing so much harm to the interests of the hospital."

### IMPORTANT TO MOTHERS.

As the result of personal experience, without any knowledge or reference to the proprietors of the article in question, and as a matter of justice to the manufacturers, we feel constrained to record our experience of Neave's food for infants. During the last three years we have watched the progress of a child deprived from birth of its mother's milk, which has been fed mainly on this food. The child has thrived exceedingly; it has made much muscle, but little fat, and is now one of the healthiest and strongest of children. The only drawback is that the first teeth have decayed rapidly, but in all other respects the results have been so satisfactory as to impel us to give this personal experience in the interest of mothers similarly situated.

